# The acceptability of, and informational needs related to, self‐collection cervical screening among women of Indian descent living in Victoria, Australia: A qualitative study

**DOI:** 10.1111/hex.13961

**Published:** 2024-01-07

**Authors:** Ana Machado Colling, Nicola S. Creagh, Neha Gogia, Kerryann Wyatt, Claire Zammit, Julia M. L. Brotherton, Claire E. Nightingale

**Affiliations:** ^1^ Centre for Health Policy, Melbourne School of Population and Global Health The University of Melbourne Melbourne Victoria Australia; ^2^ Cancer Council Centre for Behavioural Research in Cancer Melbourne Victoria Australia

**Keywords:** acceptability, cervical cancer, cervical screening, Indian women, self‐collection, self‐sampling, South Asian

## Abstract

**Background:**

In July 2022, self‐collection became universally available as part of Australia's National Cervical Screening Program. This change aims to address screening inequities experienced among underscreened populations, including women of Indian descent. This study explored experiences of cervical screening, alongside the acceptability of self‐collection, among women of Indian descent living in Victoria, Australia. We also aimed to articulate the informational needs to promote self‐collection among this population.

**Methods:**

Five focus group discussions with 39 women living in Victoria were conducted in English (*n* = 3) and Punjabi (*n* = 2). Transcripts were thematically analysed, as informed by the Theoretical Framework of Acceptability.

**Results:**

Women were motivated by the choice to self‐collect, perceiving the ability to maintain modesty and greater autonomy as key enablers. Healthcare practitioners were seen as central in supporting patient‐centred models of care. Perceived barriers to self‐collection included concerns around its accuracy and women's confidence in collecting their own sample. Widespread dissemination of culturally tailored promotion strategies communicating concepts such as 'privacy' and 'accuracy' were suggested by women to promote self‐collection.

**Conclusion:**

Self‐collection was highly acceptable among women of Indian descent, particularly when assured of its accuracy, and sociocultural norms and previous screening experiences are considered. This study highlights the huge potential that self‐collection can play in increasing equity in Australia's cervical screening programme.

**Patient or Public Contribution:**

Members of the public were involved in focus group discussions. Findings were summarised and disseminated via a poster. A bicultural worker was involved in all stages of the research.

## 1 INTRODUCTION

Australia is well positioned to become one of the first countries to eliminate cervical cancer as a public health problem (incidence of ≤4 cases per 100,000 women).^1^ Australia's achievements stem from the implementation of two strategies, a National Cervical Screening Program, implemented in 1991, and a National Human Papillomavirus (HPV) Vaccination Program, implemented in 2007.^2^ The screening programme, in particular, has resulted in a 50% reduction in cervical cancer incidence and mortality.^3^

Cervical screening in Australia is most commonly provided during consultations with a general practitioner (GP) or specially qualified nurse. Until December 2017, cervical screening was provided with cytology, using a speculum examination to visualise the cervix and take a cervical sample. In December 2017, Australia renewed its national programme, replacing 2‐year cytology with 5‐year HPV‐based screening, which provided the opportunity to introduce HPV testing of samples obtained via vaginal self‐collection (hereafter referred to as self‐collection). Self‐collection was initially available in a restricted form for underscreened or never‐screened women and people with a cervix who declined a practitioner‐collected test.^3^ The restricted pathway created substantial challenges and resulted in low uptake among those who were eligible (<0.1%, *n* < 6000).^3^, ^4^, ^5^, ^6^ In July 2022, based on strong evidence of the equivalent sensitivity between self‐collection and practitioner collection for the detection of CIN2+, Australia's programme updated its policy and made self‐collection available as a choice for everyone due for screening.^3^, ^7^, ^8^ This is still delivered through a practitioner‐supported model, where the self‐collection swab and instructions are provided by a general practitioner or a specially trained nurse within a clinical consultation, with the option for providers to collect the vaginal sample without a speculum.^3^

Despite Australia's achievements towards the elimination of cervical cancer, inequities persist, with 72% of cervical cancer cases occurring in women who are underscreened or never screened.^9^ People born in India represent 2.3% of the Australian population^10^ and are one of the fastest growing populations of people born overseas.^11^ While participation in cervical screening by ethnicity is not routinely collected at a national level,^3^ findings from a data linkage study in Victoria suggested that age‐adjusted screening rates for Indian‐born women were ~10% lower compared to Australian‐born women.^12^ Globally, there is evidence to suggest that migrant women are less likely to benefit from cervical cancer prevention programmes because of reported economic‐, cultural‐, language‐, health systems‐ and patient‐level barriers, with migrants from Asia having lower adherence to cervical screening.^13^ Barriers to cervical screening programmes amongst Indian‐born women stem from an intersection of personal,^14^, ^15^, ^16^ practical^17^ and cultural barriers,^18^ including an unfamiliarity with the concept of preventative health,^19^, ^20^ English proficiency, fatalistic attitudes,^21^ modesty and stigma associated with cancer.^20^ In addition to screening inequities, the implementation of HPV vaccination programmes varies internationally, with 70% of girls residing in countries without a nationally funded programme, including in India.^22^ Australia's HPV vaccination programme is primarily delivered through a school‐based model. As many migrant women arrive in Australia after school age, they may be ineligible for publicly funded HPV vaccination and, as a result, likely to remain unvaccinated. A study among young women in Australia found that country of birth other than Australia to be a predictor of lower uptake and completion of HPV vaccination.^23^ This highlights the ongoing importance of secondary prevention strategies, such as cervical screening.

The availability of self‐collection provides an opportunity to increase equity by mitigating many barriers to screening by increasing choice.^14^, ^15^, ^16^, ^19^, ^24^, ^25^, ^26^ International evidence demonstrates that self‐collection can increase participation among underscreened and never‐screened populations^7^, ^27^ and is highly acceptable.^28^, ^29^, ^30^, ^31^, ^32^ It affords women greater privacy, comfortability^26^, ^33^ and convenience^29^, ^33^ compared to a practitioner‐collected cervical screening test.^31^, ^34^ The major concerns people considering self‐collection consistently report relate to correctly administering the test and the accuracy.^14^, ^19^, ^28^, ^29^, ^35^, ^36^ Overall, self‐collection remains poorly promoted among population groups in Australia and is currently underutilised.^4^, ^5^, ^34^

This study aimed to understand the experiences of cervical screening, alongside the acceptability of self‐collection among women of Indian descent living in Victoria, Australia. We also aimed to articulate the informational needs to promote self‐collection among this population. Exploring the potential of self‐collection will likely contribute to enhancing participation and equity in Australia's national programme, especially among underscreened groups.

## 2 METHODOLOGY

### 2.1 Theoretical approach

This study adopted the Theoretical Framework of Acceptability (TFA)^37^ to explore the prospective and experienced acceptability of self‐collection from the perspectives of women of Indian descent. The TFA has seven domains that contribute to the acceptability of an intervention. These domains were operationalised in the context of this study (Table 1). A qualitative understanding of the prospective acceptability can highlight sociocultural or religious aspects of screening options that require more information, promotion, capacity building, self‐efficacy, modification or tailoring to the needs of women of Indian descent to increase acceptability and thus their participation in screening. Additionally, assessment of experienced acceptability can provide insight into barriers and enablers to current screening participation.

**Table 1:** Theoretical framework of acceptability as operationalised in the context of this study.

Construct	Theoretical framework of acceptability definition^37^	Operationalisation of framework definitions in the context of this study	Example questions
Affective attitude	How an individual feels about an intervention	How women feel about cervical screening and self‐collection, including previous screening experiences	Would anyone like to share their experiences with cervical screening? What are your feelings towards self‐collection?
Burden	The perceived amount of effort that is required to participate in the intervention	The perceived amount of effort, including barriers and enablers, that is required to participate in cervical screening and self‐collection	What might stop you from doing a cervical screening test? What might encourage you to do a self‐collection test?
Ethicality	The extent to which the intervention has good fit with an individual's value system	The extent to which cervical screening and the option of self‐collection is perceived to be a good fit with women's value systems, including sociocultural factors	Do you think cervical screening is something that women in your community do regularly? Why or why not? Would you speak to family and friends about cervical screening?
Intervention coherence	The extent to which the participant understands the intervention and how it works	The extent to which women understand the purpose of screening and self‐collection, including how and where the self‐collection sample is collected	Have you heard of the option to self‐collect your own cervical screening test? What other information would you like to know about self‐collection?
Opportunity costs	The extent to which benefits, profits or values must be given up to engage in the intervention	The extent to which opportunity costs (i.e., time, money, competing demands) exist to participate in cervical screening and self‐collection	What might stop you from doing a self‐collection test?
Perceived effectiveness	The extent to which the intervention is perceived as likely to achieve its purpose	The extent to which cervical screening and self‐collection is perceived to be a beneficial and effective preventative health measure to women	Can anyone tell me what they know about cervical screening? What do you think your community needs to know about self‐collection to be comfortable using it?
Self‐efficacy	The participant's confidence that they can perform the behaviour(s) required to participate in the intervention	Women's confidence in participating in cervical screening, including their confidence in performing their own sample	After hearing some information about self‐collection, would you choose this option? Why/why not? What are some of the advantages/disadvantages of the different tests we talked about today?

### 2.2 Study design

Semistructured focus group discussions (FGDs) were conducted with women* of Indian descent aged 25–74 years living in Victoria, Australia. Informed by experience in engaging and working with people of Indian descent, we chose to use gendered language (i.e. women), believing that it would be more meaningful to the population we were engaging. We chose to conduct FGDs as this enables individuals to explore and build on the responses of other group members, which may result in insights and perspectives that might not emerge in individual interviews.^38^ An experienced bicultural facilitator was present in all FGDs to support discussion among participants. FGDs comprised women from the same cultural background, stratified by community group, age and language preference where possible.^38^ A systematic review^28^ of qualitative studies exploring the acceptability of self‐collection found that most studies (56%) employed FGDs. The research team included a bicultural worker N. G. of Indian descent with medical training, existing relationships with Indian communities in Victoria and substantial experience and training in qualitative research. Eligible women were of Indian descent, aged 25–74 years, able to communicate in either English or in Hindi or Punjabi and resident in Victoria, Australia. This paper is reported according to the consolidated criteria for reporting qualitative research (COREQ) guidance.^39^

### 2.3 Recruitment

A flexible recruitment design using convenience sampling techniques was employed. This involved author N. G. leveraging connections with community members and leaders to engage with and recruit women. Author N. G. approached two community leaders who invited 15 women each from their community centres. Word of mouth by community members who received the advertisement were invited to distribute the information to other eligible women as a snowballing recruitment approach. Women were invited to the study by (1) targeted email and SMS messages circulated in English or Hindi and (2) advertisement via a social media platform (Instagram). Author N. G. collected contact details from interested participants and forwarded an online registration survey, plain language statement and written consent form to complete before attending the FGD. Participants were asked to indicate their preferred dates and time for Zoom FGDs. Women received a $100 gift card as an acknowledgement of their participation.

### 2.4 Data collection

Semistructured FGDs using open‐ended questions were co‐facilitated via Zoom by author A. M. C. (female researcher) and the bicultural worker N. G. The FGDs were conducted in English or in a language as preferred by women, as informed by N. G. For in‐language FGDs, N. G. facilitated and A. M. C. provided technical support. The FGDs covered themes including perceptions and experiences with cervical screening, attitudes about self‐collection and strategies to promote self‐collection to women of Indian descent (Table 1). Due to women's low awareness of self‐collection, an overview and a visual guide demonstrating how to use the self‐collection swab was provided during FGDs. Before data collection, the FGD guide was piloted with bicultural workers at a Victorian community‐based organisation and two Indian Hindi‐speaking researchers in the broader research team to ensure cultural appropriateness, the flow of questions, visual content and duration of discussion.

### 2.5 Analysis

All FGDs were audio recorded and transcribed verbatim. The in‐language FGDs were professionally translated to English before verbatim transcription and cross‐checked by N. G. for accuracy. Deductive thematic analysis using an a priori coding framework informed by the TFA was employed and analysed using NVIVO 12. After familiarisation with the data, an inductive approach was applied to identify emerging subthemes within the higher level themes of the framework to reflect accuracy and depth of the data. Coding was done by A. M. C. and discussed at length throughout analysis with N. S. C. and C. E. N. until no new themes emerged (saturation).^40^ After a preliminary analysis, N. G. provided cultural contextualisation and supported the interpretation of analysis. Further analysis exploring differences in self‐collection acceptability between women who were overdue/never screened and women who were up to date with screening yielded no observable differences. Socioeconomic status was derived from participants' postcodes and categorised into five quintiles (low, low‐medium, medium, high‐medium and high) of the Australian Bureau of Statistics data Index of Relative Socio‐Economic Advantage and Disadvantage (IRSAD Deciles) for Statistical Level Area 2.^41^

### 2.6 Research team and reflexivity

The research team acknowledges the importance of the principle of reflexivity. The first author, A. M. C., as a non‐Indian woman, kept a record of personal reflections, contextualisation and interpretation towards the data collection and analysis. Author N. G. is an Indian woman and has extensive experience working with Indian and other culturally and linguistically diverse communities. Other authors (N. S. C., C. E. N., J. M. L. B., C. Z., K. W.), as non‐Indian people, hold various roles within cancer screening research and promotion, with a focus on underscreened populations to improve equity in healthcare access and practice reflexivity within their roles.

## 3 RESULTS

A total of 40 women were invited to participate in the study, of whom 39 (98%, *n* = 39) consented to participate (Table 2), with one woman not responding to the invitation. Five semistructured FGDs were carried out between 24 July and 30 July 2022. These discussions were conducted in English (*n* = 3) and Punjabi (*n* = 2), with seven to nine women in each group. The FGDs lasted between 73 and 86 min, with a mean duration of 79.8 min. Women's age ranged between 27 and 71 years, (mean = 44.1 years, median = 39 years). Most women (64.1%, *n* = 25) resided in areas with a medium category socioeconomic status. All women were born in India, with the majority (85%, *n* = 33) having lived in Australia for more than 5 years and (97%, *n* = 38) spoke more than one language. Most women had previously had a cervical screening test (67%, *n* = 26), and of these five women (19%) were overdue according to the national guidelines. Of those who had previously had a cervical screening test, 25 women (96%) reported that this was done in Australia, with one woman reporting completing cervical screening in another country. Table 2 describes the demographic characteristics of women.

**Table 2:** Characteristics of 39 Indian‐born women participating in focus groups on self‐collection in July 2022, Victoria, Australia.

Category	Variable	Number (*n* = 39)	%
Age	25–49 years^a^	30	77
	50–74 years	9	23
Postcode	Metropolitan	36	92
	Rural	3	8
Socioeconomic status	Low	8	20
	Low‐medium	1	3
	Medium	25	64
	High‐medium	4	10
	High	1	3
	Total	39	100
Country of birth	India	39	100
Length of time in Australia	<5 years	6	15
	5–10 years	10	26
	10+ years	23	59
Spoken language(s)	English	22	56
	Punjabi	34	87
	Hindi	39	100
	Other	4	10
Cervical screening participation	Yes—up to date	21	54
	Yes—not up to date	5	13
	No	13	33
Place of last screening test (*n* = 26)	Australia	25	96

^a^ Women under 30 years (*n* ≤ 5). Specific numbers are not included to ensure confidentiality.

**Figure 1:**
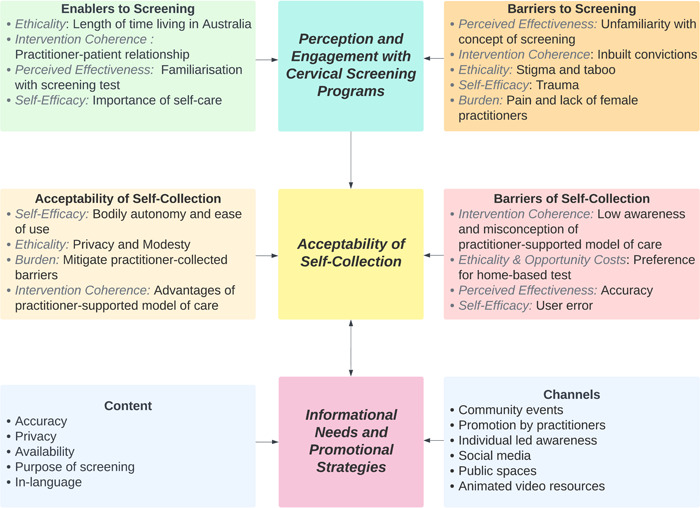
Overview of results from focus group discussions with 39 Indian‐born women on their experiences with cervical screening and their perceptions, acceptability and informational needs of self‐collection cervical screening with subthemes aligned to the Theoretical Framework of Acceptability constructs.

Results are presented in three main themes: (1) perceptions and engagement with cervical screening; (2) the acceptability of self‐collection and (3) the informational needs and appropriate health promotion strategies. Figure 1 provides an overview of the study's key themes and their connection to the TFA constructs.

### 3.1 Perceptions of and engagement with cervical screening programmes

Women's previous experience with cervical screening and the role of Indian sociocultural norms influenced women's decision to participate in screening and how they perceived the acceptability of self‐collection.

Familiarisation with the practitioner‐collected cervical sample enhanced by women's length of stay in Australia and practitioners' expertise were enablers for screening participation. These enablers were important for integrating the ethicality of screening into women's individual value systems and improving women's intervention coherence and experiences with cervical screening. Women spoke of the importance of taking onus of their own health, illustrating the role of self‐efficacy for regular screening participation.

… encouragement is mainly especially when you're new to this country, and you haven't heard about all these tests in your home country. And you will feel this is something really new and important that doctor is telling you the first time is very prompt, everyone, whenever they hear about it, and they get to know it, this is something women should get it done, they get it done. [*Participant 1, FGD 2, 41 years*]

However, values and traditional beliefs prevalent in Indian culture, including stigma towards discussing sexual health, and an unfamiliarity with and inbuilt convictions about preventative health influenced low awareness and intervention coherence towards screening. Some women perceived screening as ‘unnecessary' in the absence of symptoms.

Aligned with cultural values, modesty represented a significant barrier, with participants describing the practitioner‐collected cervical screening as ‘*embarrassing*’, ‘*awkward*’, ‘*uncomfortable*’ and ‘*painful*’ with the majority of women considering this a burden to participate in cervical screening. These negative experiences and perception of limited access to female practitioners in Australia impacted women's self‐efficacy to rescreen.

I find myself very uncomfortable with having the Pap smear done. So that is why I think it's the same reason that I don't feel like going [to have a cervical screen]. Because whenever I had done that test [practitioner‐collected cervical screening], I always feel uncomfortable. And it [practitioner‐collected cervical screening] is painful for me. [*Participant 12, FGD 2, 41 years*]

### 3.2 Acceptability of self‐collection cervical screening

Overall, women were motivated by self‐collection, seeing it as an opportunity for greater autonomy and increased modesty.

Most women (*n* = 29, 74.4%) were not aware of self‐collection before attending the FGDs and very few had any knowledge of self‐collection, with the exception of three (8%) women who had previously performed self‐collection, describing the ethicality of maintaining modesty (Quote_1.1). After a description of self‐collection was provided to women during the FGDs, women were supportive of self‐collection and perceived it as an ‘*encouraging*’ and ‘*convenient*’ option. Self‐collection was perceived to afford women greater privacy, comfort, bodily autonomy and agency (Quote_1.2). These anticipated advantages created a positive affective attitude and greater self‐efficacy afforded by self‐collection to mitigate previously described personal barriers with practitioner‐collected cervical screening. For some women, self‐collection was perceived to address practical barriers to screening, such as the need for female practitioners (Quote_1.3). This minimised the amount of perceived effort to participate in screening, thus reducing its burden (Table 3).

**Table 3:** Acceptability of self‐collection with supporting quotes.

Subtheme	Construct	No.	Quotes
Experienced acceptability of self‐collection	Ethicality	1.1	‘… that stigma of somebody's looking into my vagina is not there so that thing is the most important thing you know? And yeah, and it [self‐collection] is nothing much, it [self‐collection] is an easy thing to do’. [*Participant 33, FGD 5, 35 years*]
Anticipated acceptability of self‐collection	Affective attitude	1.2	‘it's always good to do it on your own, like more privacy and respectful way maybe … When you can do it by yourself why depend on somebody else?’. [*Participant 36, FGD 5, 40 years*]
Ability of self‐collection to mitigate barriers associated with a practitioner‐collected cervical sample	Burden	1.3	‘… we do nasal swabs and other swabs as well, why not a vaginal swab? And if it [self‐collection] is detecting the virus, it's a positive approach towards a more comfortable environment than going to a GP and looking for a female doctor all the time, because the male GP can easily explain the instructions and wait time would be a lot lesser than what we always fear of booking an appointment, I guess. So definitely. It [self‐collection] will reduce the burden and make more women aware to come forward and do this test’. [*Participant 11, FGD 2, 33 years*]
Confidence in administrating own test	Intervention coherence	1.4	‘Once we have some knowledge, and we feel comfortable from our experience, we can educate others it is ok to use self‐collection method as well’. [*Participant 27, FGD 4, 34 years*]
Role of practitioner in promoting self‐collection	Self‐efficacy	1.5	‘… Once you trust yourself, then you can actually do it [collect your own vaginal sample]. Maybe one time, definitely everyone would like to go to GP, and then, at that time, when GP is performing [assisted vaginal swab], they can explain in such a way so that the other person can feel they can do it [self‐collection] next time by themselves. Yes, definitely, you know, because the only way of showing practically is by performing, so maybe after one performance, they can realize, okay, I can just do it myself from next time’. [*Participant 25, FGD 4, 31 years*]

Women were optimistic that once they try self‐collection and developed greater intervention coherence of this new screening option, they would be able to advocate to other women in their community (Quote_1.4). For women who expressed enthusiasm towards self‐collection, it was clear that health practitioners still played an influential role in promoting confidence and intervention coherence in the pathway. For some women, this meant feeling more comfortable with an initial practitioner‐collected vaginal sample and opting to self‐collect next time they were due for screening (Quote_1.5).

Despite the perceived advantages of self‐collection, women held a general misconception that self‐collection would be a home‐based test, rather than a test provided within the practitioner‐supported delivery model that exists in the Australian programme. Older women expressed the ethicality of collecting the sample at home, by removing the cultural stigma of discussing and performing an intimate test near other males in a primary care setting (either practitioners or community members). Women noted the time, effort and costs required to attend a face‐to‐face consultation with a practitioner, highlighting the opportunity costs associated with Australia's practitioner‐supported model of care.

The biggest advantage of doing the self‐collection at home is it can provide people with a safe and secure environment to collect the sample, because a lot of people in our community feel a little shy about going to a GP clinic. Oftentimes we also see a lot of men at a GP clinic. At home you can just relax and take the sample at your own time, while at the GP clinic, they might provide you with a male assistant, which can be a little inconvenient. [*Participant 22, FGD 3, 66 years*]

Despite most women seeing self‐collection as an opportunity for greater autonomy, concerns emerged around the novelty of, perceived accuracy and user error influencing the perceived effectiveness of self‐collection. This resulted in some women having greater confidence and trust in a practitioner performing the test, including a practitioner‐assisted vaginal sample. Women, who initially indicated they would prefer self‐collection if they were able to perform the test at home, stated they would prefer a practitioner‐collected cervical or vaginal sample if they had to attend an appointment. Women raised concerns around correctly collecting the self‐collection swab, with this lack of self‐efficacy leading to women fearing incorrect results due to user error. The fear of a false‐negative result was further exacerbated in the context of the renewed 5‐year screening interval timeframe. Further, aligning with sociocultural norms reported in Theme 1, cultural shyness towards discussing cancer and intimate body parts was reported as an inhibitor to women's confidence to self‐collect.

… just in general uncomfortable to be doing it [self‐collection] and then you know, this trust factor as if you know, I've done it right or not done it right. [*Participant 8, FGD 1, 36 years*]

### 3.3 Informational needs

To create community awareness, women reported that they, first, require an awareness that self‐collection is an available option.

… we need to convey the message that self‐collection is the new and readily available option that be done under the supervision of the GP to more and more people in the community … There is no awareness about the necessity and importance of these [cervical screening] tests. [*Participant 17, FGD 3, 70 years*]

In‐language and plain language materials communicating the purpose and importance of screening in preventing cervical cancer and the accuracy and ease of self‐collection emerged as important informational needs for women of Indian descent. Highlighting that self‐collection affords women privacy was perceived as central in promoting screening among women of Indian descent, as it aligned with their ethicality around modesty that is often inhibited by a practitioner‐collected CST.

Most important flashlight, if you really want to get through the community, is like, it [self‐collection] is private, you can do it by your own. [*Participant 33, FGD 5, 35 years*]

Women provided suggestions for how self‐collection could be promoted. Women believed that practitioners played a key role in promoting self‐collection to their patient group. Women also believed that promotion within the community, by the community, at events would be a useful strategy. Many women perceived the use of animated video resources as a practical way of demonstrating how self‐collection works and a way of building women's confidence to collect the swabs themselves. Women expressed a desire for pop‐up clinics, where they could access information on cervical and breast cancer and other health‐related issues of women. Other promotional channels suggested included community radio, community centres, public toilets, healthcare clinics, shopping centres, petrol stations, religious places, libraries, postal and SMS reminders, government health websites, television, council advisory groups, social media and within schools and universities.

And it [promotional campaign] will definitely improve knowledge. And definitely encourage people to go for self‐test or book an appointment, discuss with the doctor, that will, that will remove their, you know, fear, I think their inhibition or lack of knowledge as well, I think this platform should be used, either through Instagram, or Facebook, or YouTube. [*Participant 5, FGD 1, 41 years*]

## 4 DISCUSSION

To our knowledge, this is the first qualitative exploration of the anticipated or experienced acceptability of and informational needs for how best to implement the universal choice of self‐collection among women of Indian descent living within Australia. Findings highlight how sociocultural norms and previous screening experiences influence women's choices in participating in cervical screening. Despite initial low awareness, self‐collection was perceived as highly acceptable, as a new opportunity to increase autonomy when screening, to maintain modesty and to mitigate barriers associated with a pelvic examination. However, the acceptability of self‐collection was hindered by concerns about accuracy and misconceptions that it was a home‐based test. There was an unmet need for in‐language resources within primary care and culturally tailored health promotion strategies that communicate the accuracy, ease of use and privacy of self‐collection to increase acceptability. Women's unfamiliarity with the concept of preventative health and the perception that asymptomatic women are at low risk of cervical cancer echoes previous studies on the role of cultural practices and assumptions in shaping knowledge, awareness and engagement of screening programmes.^19^, ^20^, ^21^, ^42^ Similarly, a survey of 316 health practitioners in India found the most significant barrier to screening among their patient group was being asymptomatic, followed by embarrassment and fear of cancer diagnosis, and thus, there is a need to raise awareness to debunk myths and clarify facts related to cervical screening and HPV vaccination.^43^

A systematic review in India exploring the implementation of self‐collection found patient‐level barriers, including lack of knowledge and misconceptions, low prioritisation to screen and poor sample quality due to a lack of self‐efficacy outweighed provider‐ and systems‐level barriers.^44^ Solutions to address patient‐level barriers centred around community engagement and education strategies, including access to self‐collection swabs for women to develop familiarisation with the test and to mitigate fears and misconceptions related to self‐collection.^43^ These studies provide contextualisation to our findings on barriers to women's screening participation and views of self‐collection in Australia. Despite these barriers, studies have demonstrated high acceptability of self‐collection for cervical screening among women in India.^45^, ^46^, ^47^, ^48^, ^49^

Overall, women in the present study were highly supportive of self‐collection, citing greater privacy, increased modesty, comfort, convenience and bodily autonomy as advantages. These findings align with studies exploring self‐collection among the general Australian population who are underscreened or never screened, who reported that it was a highly acceptable and an empowering screening option.^3^, ^19^, ^25^, ^50^, ^51^ Of note, many women in this study reported that they would be more comfortable with having a practitioner collect the vaginal sample with the self‐collection swab, particularly the first time they used this method. This option was thought to alleviate barriers associated with the speculum examination, while at the same time affording women the confidence of a practitioner‐collected sample. This preference was also reported among culturally and linguistically diverse women in a study conducted in the United Kingdom and highlights the need for practitioners to present all screening options in a patient‐centred way.^52^, ^53^ Similarly, a study of practitioners in Victoria who were early adopters of self‐collection reported that patient‐centred approaches when offering self‐collection to underscreened or never‐screened participants were central to its uptake.^54^

Women in this study expressed a preference for home‐based models of self‐collection to further increase privacy and cultural appropriateness. Current national screening guidelines recognise the need for practitioners to offer flexible and patient‐centred models of care and allow collection at home or out of the clinic as long as appropriate clinical support is available.^3^ This has been trialled through telehealth models during the pandemic^55^ but could also be achieved through home visits, supported through women's group events with practitioner supervision or by allowing the woman to take the swab home as long as a system is in place to return it for processing. A recent randomised controlled trial in the United States found mailed‐out self‐collection kits increased uptake of screening but reported on low rates of follow‐up attendance.^56^ More investigation is needed to evaluate the effectiveness of these differentiated models on cervical screening uptake in Australia.

Although almost half the women in this study were eligible for self‐collection, very few were aware of its availability, underscoring that it has been poorly promoted and underutilised.^5^, ^34^ This low awareness appeared to have a negative impact on the acceptability of self‐collection, with few women having a correct understanding about how to access the test, or surrounding accuracy.^7^ Further impacting women's acceptance of self‐collection was a concern about collecting the sample correctly. These concerns have been well described in the literature;^14^, ^19^, ^28^, ^29^, ^35^, ^36^, ^57^ yet, studies have found this concern to be alleviated once women perform the test.^25^ Moreover, Australian literature considering the practitioner's perspective on the implementation of self‐collection reported that its limited adoption in clinical practice is in part due to lack of clear communication and promotion.^3^, ^6^, ^54^, ^58^, ^59^ The practitioner‐supported model of care in Australia provides an opportunity for practitioners to accurately explain these features and to demonstrate how to accurately collect the sample. Ensuring practitioners are informed and confident regarding self‐collection is imperative, considering a trustful relationship is needed to mitigate known barriers of cervical screening and self‐collection.

This study emphasises the need for, and importance of, in‐language resources delivered by trusted sources to increase community awareness of self‐collection among women of Indian descent. This requirement has also been identified by health practitioners with experience providing cervical screening for culturally and linguistically diverse populations in Australia.^60^ A scoping review in Australia confirmed inadequate support for both culturally and linguistically diverse patients and practitioners across the cancer care continuum and calls on structural and health system reforms to provide culturally and linguistically responsive and equitable services.^61^ A large suite of in‐language resources are available in Australia's National Cervical Screening Program^62^, ^63^, ^64^ however, ensuring resources are up to date with self‐collection information and meet the language and informational needs to address specific cultural barriers and enablers for behavioural change of all culturally and linguistically diverse populations in Australia is critical. Many of the informational resources suggested by women in this study already exist; yet, women were not accessing them, suggesting that current channels are not reaching the target population.

A wide range of promotional platforms were suggested to disseminate culturally tailored messages to increase awareness and knowledge of self‐collection. A qualitative study among GPs in Australia suggested the use of personalised in‐language SMS reminders as more effective than invitation letters for cervical screening participation.^60^ Similarly, in‐language phone calls have been found to be more effective in increasing participation in Australia's breast screening programmes.^65^ These findings align with the evidence base informing Australia's draft national elimination strategy to co‐develop targeted and widespread campaigns and informational resources that are responsive to the needs of priority communities.^66^ Delivering cervical screening information across a range of channels has found to increase participation among culturally and linguistic populations.^67^

## 5 STRENGTHS AND LIMITATIONS

This study has several strengths, including the high response rate (97.5%, *n* = 39/40) of women. The utilisation of the TFA highlighted aspects of self‐collection and screening more broadly that require more information, promotion, capacity building, self‐efficacy, modification and tailoring to the needs of women of Indian descent to increase acceptability and participation. The involvement of a bicultural worker N. G. throughout the recruitment, data collection and analysis ensured the research was carried out in a culturally appropriate manner. The study design further allowed the research team to answer women's questions about cervical screening and self‐collection and clarify misconceptions in real time.

This study sample was limited to those living in the Australian state of Victoria. This provided the research team with confidence that findings reflected the depth of attitudes in local communities within Victoria, rather than trying to capture differences on a national level that would require a sampling framework outside the scope of this study. The views of younger Indian women (25–30 years old) who are eligible for self‐collection for the first time since universal access is scant in our data. Despite the advantages of qualitative research, the authors acknowledge that participants' responses might have been impacted by the presence of social desirability bias.^68^ This study was restricted to English‐, Hindi‐ and Punjabi‐speaking women. Given the ethnic diversity prevalent in India, future research with other ethnic groups is warranted. Further, this study only included people from an Indian descent who identified as women, and as such, future research should capture the nuanced perspectives of cultural and linguistically people with diverse gender identities. Lastly, many women in this sample have been living in Australia for 10 or more years (*n* = 23, 59%), and as a result, future research should also consider the perspectives, experiences and needs of more recently arrived migrants.

## 6 CONCLUSION

Australia is on track to eliminate cervical cancer within the next decade.^69^ As the roll out of universal self‐collection continues, it is timely to investigate the needs of populations who are underscreened to inform the successful implementation of this new pathway for all groups within Australia. This study provides an in‐depth and theoretically informed understanding of the anticipated and experienced acceptability of self‐collection among women of Indian descent in Victoria and identifies future implementation efforts that can be leveraged to support equitable participation in screening: First, widespread dissemination of messages that communicate the purpose of screening alongside keywords such as modesty, availability and accuracy across promotional channels to increase women's awareness of and comfort with the use self‐collection and mitigate misconceptions is encouraged. Second, tailoring and providing existing in‐language resources to trusted sources, such as practitioners and bicultural health workers and prominent community leaders. Last, ensuring practitioners know they can offer patient‐centred flexibility in their delivery of cervical screening, including offering to collect vaginal samples and facilitating at‐home collection when this is strongly preferred by the woman.

## AUTHOR CONTRIBUTIONS

**Ana Machado Colling**: Conceptualisation; methodology; formal analysis; writing—original draft; writing—review and editing; data curation; investigation. **Nicola S. Creagh**: Conceptualisation; methodology; formal analysis; supervision; writing—review and editing. **Neha Gogia**: Formal analysis; writing—review and editing; investigation; data curation. **Kerryann Wyatt**: Conceptualisation; writing—review and editing; supervision. **Claire Zammit**: Writing—review and editing; conceptualisation. **Julia M. L. Brotherton**: Conceptualisation; methodology; writing—review and editing; supervision. **Claire E. Nightingale**: Conceptualisation; funding aquisition; methodology; formal analysis; writing—review and editing; supervision.

## CONFLICT OF INTEREST STATEMENT

J. M. L. B. was previously employed by ACPCC. ACPCC has received donations of equipment and HPV test kits from Roche, Seegene, Abbott, BD, Cepheid and Copan for research and validation studies.

## ETHICS STATEMENT

Ethics approval was gained from the University of Melbourne, Medicine and Dentistry Human Research Ethics Sub‐committee (Ethics ID: 23536). Participants gave informed consent to participate in the study before taking part. Informed written consent was obtained from all women involved in this study.

## Data Availability

The data that support the findings of this study are available on request from the corresponding author. The data are not publicly available due to privacy or ethical restrictions.

## Footnotes

* Recognising that not all people requiring cervical screening identify as women and may identify as ‘people with a cervix’. The research team at the University of Melbourne, in conjunction with Cancer Council Victoria, considered this point heavily. It was the opinion of Cancer Council Victoria, which has considerable experience engaging and working with people of Indian descent populations, that terminology such as ‘people with a cervix’ would not be meaningful to this population. It is for this reason that this study has used gendered language.
